# Comparison of In-Solution Biorecognition Properties of Aptamers against Ochratoxin A

**DOI:** 10.3390/toxins8110336

**Published:** 2016-11-15

**Authors:** Maureen McKeague, Ranganathan Velu, Annalisa De Girolamo, Stefania Valenzano, Michelangelo Pascale, McKenzie Smith, Maria C. DeRosa

**Affiliations:** 1 Chemistry Department, Carleton University, 1125 Colonel by Drive, Ottawa, ON K1S 5B6, Canada; mmckeague@gmail.com (M.M.); rvelunathan@gmail.com (R.V.); mckenziesmith@cmail.carleton.ca (M.S.); 2 Institute of Sciences of Food Production, National Research Council of Italy, via G. Amendola 122/O, Bari 70126, Italy; annalisa.degirolamo@ispa.cnr.it (A.D.G.); stefania.valenzano@ispa.cnr.it (S.V.)

**Keywords:** aptamer, ochratoxin A, mycotoxins, biorecognition, fluorescent assay, biosensing, aptasensor, microscale thermophoresis

## Abstract

Ochratoxin A (OTA) is a mycotoxin produced as a secondary metabolite by several species of *Aspergillus* and *Penicillium* and frequently found as a natural contaminant in a wide range of food commodities. Novel and robust biorecognition agents for detecting this molecule are required. Aptamers are artificial nucleic acid ligands able to bind with high affinity and specificity to a given target molecule. In the last few years, three separate research groups have selected aptamers for ochratoxin A. While each of these three families of aptamers have been incorporated into various methods for detecting OTA, it is unclear if each aptamer candidate is better suited for a particular application. Here, we perform the first head-to-head comparison of solution-based binding parameters for these groups of aptamers. Based on our results, we provide recommendations for the appropriate choice of aptamer for incorporation into solution-based biorecognition assays and applications.

## 1. Introduction

Ochratoxin A (OTA) is a mycotoxin produced as a secondary metabolite by several species of *Aspergillus* and *Penicillium* and frequently found as natural contaminants in a wide range of food commodities, including cereals, coffee, cocoa, wine, beer, spices, dried fruits, and grape juice [1]. OTA has been shown to be hepatotoxic, genotoxic, teratogenic and immunotoxic to several species of animals [2]. Moreover, sufficient evidence of carcinogenicity in experimental animals has prompted the International Agency for Research on Cancer (IARC) to classify OTA as a possible human carcinogen (Group 2B) [3]. International regulatory limits and guidelines have set worldwide for OTA in foodstuffs. In particular, the European Commission has established maximum permitted levels of OTA ranging from 0.5 to 20 μg/kg depending on the food matrix [4,5].

Due to its prevalence and importance, several analytical methods have been developed and extensively reviewed for the quantitative determination of OTA in foodstuffs [6,7,8,9]. These methods, which allow the detection of OTA alone or in combination with other mycotoxins, are mainly based on high-performance liquid chromatography (HPLC) coupled to fluorescence or mass spectrometry detection. Although most validated methods are chromatographic techniques, alternative strategies have been proposed; in particular, a number of promising immunochemical methods, such as enzyme-linked immunosorbent assays using monoclonal antibodies, fluorescence polarization immunoassays, lateral flow or immunochromatographic devices, flow-through immunoassays and optical or electrochemical biosensors including metal nanoparticles-based immunoassays have been reported for OTA analysis [10,11,12,13,14,15,16,17,18].

Immunochemical methods are based on the use of antibodies as receptor molecules that have high affinity and selectivity toward OTA. However, antibodies have some limitations mainly associated with their in vivo production. Therefore, alternative biorecognition agents are increasingly being employed for use in mycotoxin assays.

One novel class of receptor includes aptamers, artificial single-stranded DNA or RNA sequences that fold into secondary and tertiary structures giving them the ability to bind certain targets with equal or higher affinity and specificity than their equivalent antibodies [19,20]. Aptamers are selected by an in vitro procedure called SELEX (Systematic Evolution of Ligands by EXponential enrichment) [21,22]. Aptamers can be generated for highly toxic targets as well as for targets that do not elicit an immune response in vivo. The procedure can be automated and in general allows for more rapid and cost-effective production compared to antibodies. Importantly, aptamer sequences can be easily modified and labelled with a variety of molecules, such as enzymes, biotin, and fluorescent dyes that enable their incorporation into a variety of detection schemes [23].

In the last few years, several aptamers have been reported for mycotoxins, with OTA as the most investigated one [24]. As a result, several OTA aptamer sequences have been reported by three separate groups [25,26,27]. Briefly, the first promising aptamer candidate was a 36-mer sequence (designated as 1.12.2) with a reported dissociation constant (*K*_D_) in the nanomolar range [25]. To date, this aptamer has been integrated into several applications for OTA analysis, including colorimetric, electrochemical, electrochemiluminescent, fluorescent biosensors, enzyme-linked assays and affinity chromatography approaches [28]. Next, two other OTA aptamer sequences (designated as H8 and H12) were reported by Barthelmebs et al. [26]. This family of aptamers was used to develop competitive Enzyme-Linked Aptamer Assays for determination of OTA in wine samples [26]. The binding studies of these sequences also showed *K*_D_ values in the nanomolar range. A final family of aptamers was recently developed for incorporation into a label-free fluorescence assay. These aptamers, including one designated as A08 and the related minimal aptamer sequence A08min, also displayed *K*_D_ values in the nanomolar range [27].

**Figure 1:**
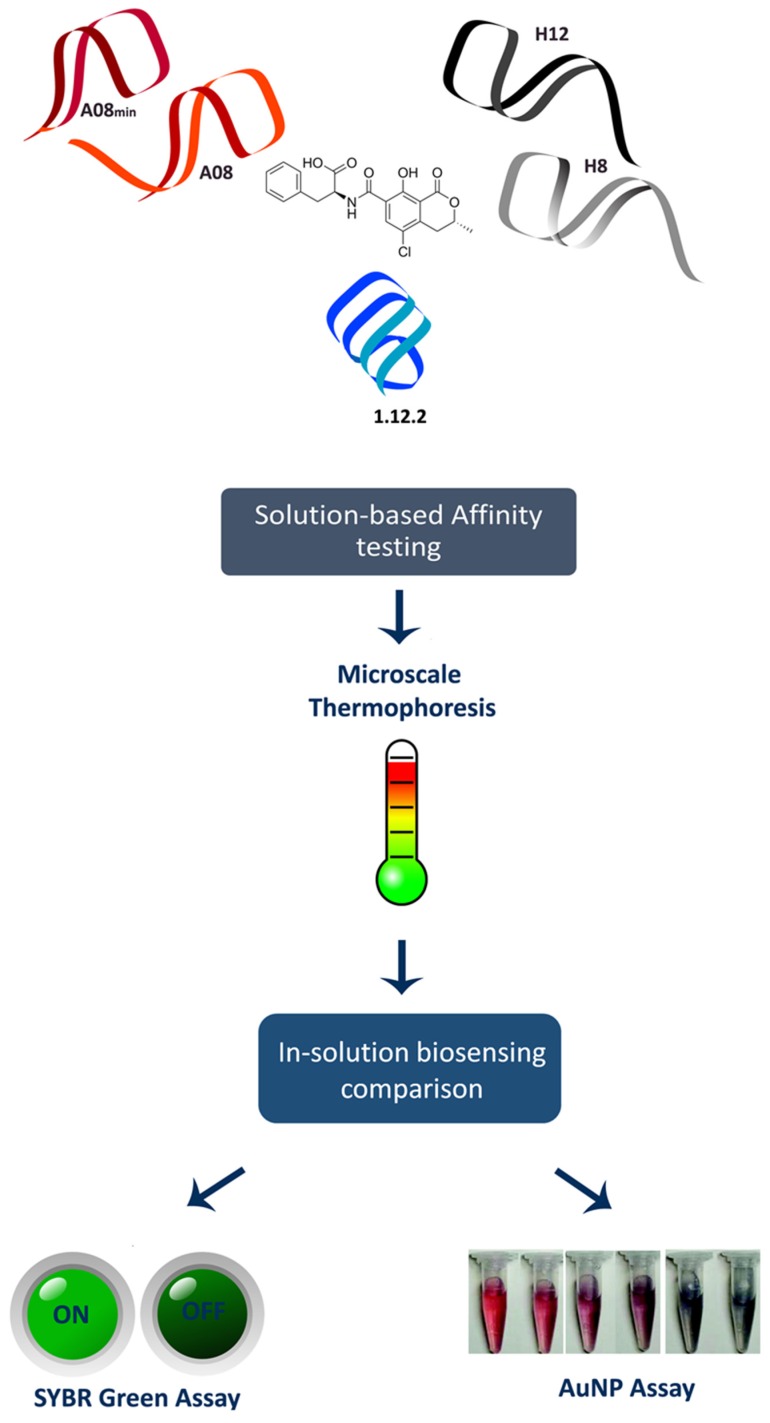
Schematic of the in-solution binding characterization of Ochratoxin A (OTA) of three families of aptamers, i.e., A08 and its minimer (A08m), H12 and H8, and 1.12.2. Microscale Thermophoresis was first used to precisely measure the binding affinities of the aptamers. Next, the in-solution biosensing abilities were assessed using the SYBR Green I Assay and gold nanoparticle (AuNP) assay.

The majority of aptamer-based OTA detection methodologies have incorporated the 1.12.2 aptamer. However, there is precedence that a given aptamer may have limitations or opportunities that make it better suited for certain molecular recognition schemes or applications. Indeed, a recent report suggests that there may be important differences beyond binding affinity (measured as the *K*_D_) between these three families [29]. Thus, the development of aptamer-based technology for OTA determination requires a thorough understanding of the opportunities and limitations for each aptamer candidate. To-date, there has been substantial efforts to test and improve the binding buffer conditions for each individual aptamer (e.g., changing the concentration of cations, pH) [30]. Despite many examples of such studies, the binding mechanisms of these three groups of OTA aptamers have not been elucidating in depth. Here, we performed the first head-to-head comparison of precise in-solution binding capabilities of the OTA aptamers by microscale thermophoresis (MST) (Figure 1). MST is a recently described method that leverages the physical phenomenon of molecular movement within temperature gradients. Each molecule (or aptamer) has a very specific “thermophoresis” that is dependent on the size, charge and hydration shell; therefore, upon binding to a target molecule, at least one of these parameters will be altered and can be measured. Because the movement of the interacting partners can be monitored with fluorescence, in-solution binding information can be obtained [31]. Following this, we then performed the first direct comparison of the analytical biosensing parameters of the three aptamer families. We performed this comparison using two separate in-solution binding assays: the SYBR Green I (SG) assay and the gold nanoparticle (AuNP) assay (Figure 1).

## 2. Results and Discussion

### 2.1. In-Solution Binding Affinity of OTA Aptamers

Previous work highlighted the importance of measuring aptamer affinity using multiple binding assays prior to integration within an application platform [29]. One important conclusion of that work was that for utilizing aptamers in common solution-based biosensing applications, the measured interaction between the aptamer and the target free in-solution is required for assessing successful aptamer performance. A major drawback of this previous work was that there was no generally applicable method for precisely measuring in-solution dissociation constants (*K*_D_ values). The SG assay did not provide precise measurements, whereas the AuNP assay could only provide qualitative binding information. However, for analytical biosensing applications accurate and precise measurements are required. To mitigate this, we employed a novel technology known as microscale thermophoresis (MST) to compare the aptamer dissociation constants directly in-solution.

**Figure 2:**
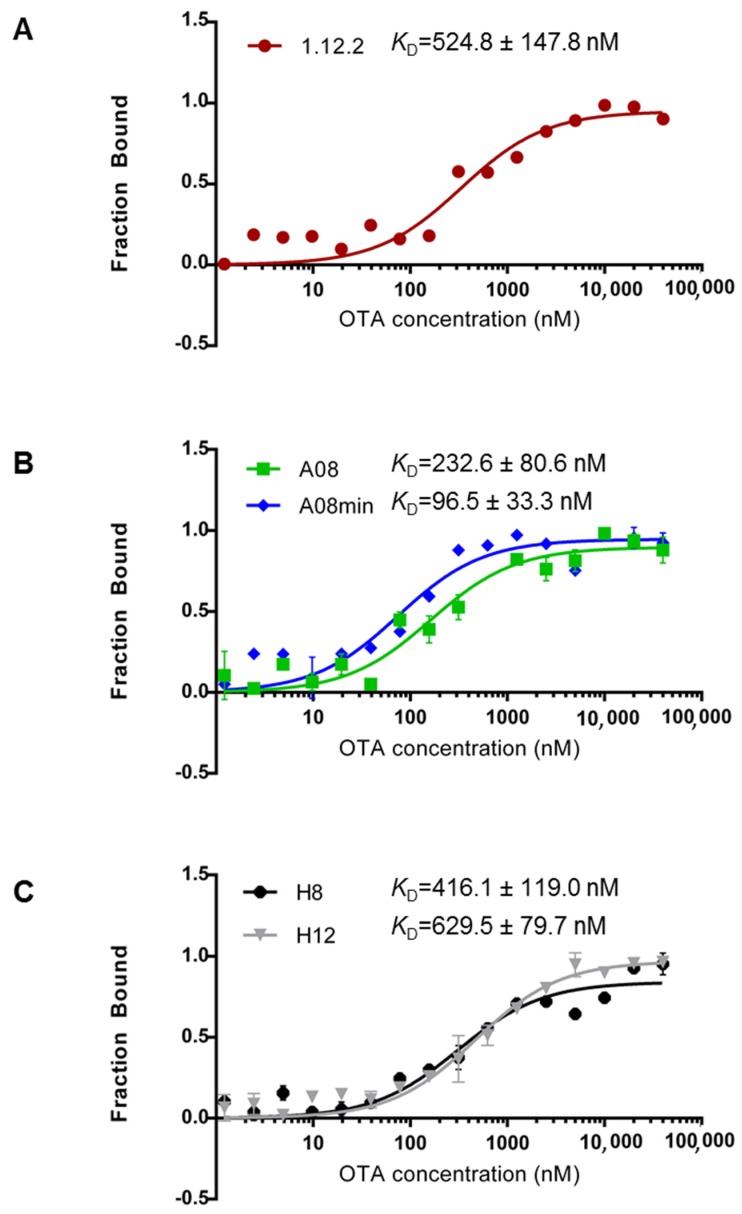
Binding isotherms and reported *K*_D_ values for each aptamer family obtained using microscale thermophoresis. (**A**) 1.12.2; (**B**) A08 and A08min; (**C**) H8 and H12. Each *K*_D_ value is the mean and standard deviation of two individual trials.

The recently described MST method is based on the characteristic thermophoretic movement of molecules through a temperature gradient. This movement is very sensitive towards changes in size, charge and hydration shell; thus, binding to a target will alter the thermophoretic behavior. The thermophoretic movement of a target molecule in thin glass capillaries through μm-sized temperature gradients can be recorded and used to determine binding interactions. As a result, the MST assay is very flexible. First, each interaction can be measured in its respective binding buffer. Secondly, the intrinsic fluorescence of the target may be used (similar to the previously employed equilibrium dialysis and ultrafiltration methods); alternatively labeling of the aptamer is possible at either the 5′ or 3′ end (similar to the surface plasmon resonance and DNAse I assays). Therefore, unlike most of the other methods employed to-date, there are currently no limitations preventing successful measurement of all aptamers in solution using this method. As a result, we were able to directly compare the in-solution binding affinity of all of these aptamers in their corresponding binding buffers using the same methodology. The sequences and respective buffers can be found in Table 1. Figure 2 highlights the measured *K*_D_ values for each aptamer using MST. From our comparison, we can clearly conclude that each of these aptamers binds with high affinity (nanomolar range) in-solution (Figure 2). Furthermore, these data compare favorably with previous reports [25,26,27,29] and confirm that all of these aptamers are good candidates for in-solution OTA biorecognition assays. Comparing these aptamer families in more detail, A08min (the A08 minimal aptamer sequence) recognizes OTA with the highest affinity and therefore may be the best aptamer choice for improving the sensitivity of OTA detection assays. This aptamer is also a desirable candidate given its shorter length (40-mer), and thus the cost of production will be lower than the A08 full length aptamer and the H8 and H12 aptamers. Aptamer 1.12.2 is another suitable candidate given its short length (36-mer); however, the affinity is more similar to all other candidates and displays approximately five-fold less affinity compared to A08min using this assay (Figure 2).

One important observation is that in contrast to most reports, the 1.12.2 aptamer displayed approximately two-fold lower affinity with this assay. A potential reason for this large inconsistency with previous reports is that the analysis for the change in signal with this aptamer was different than with A08, A08min, H8 and H12. In particular, in performing this assay, a quenching effect was detected, meaning that the fluorescence was reduced at higher levels of target. To clarify this, a denaturing test (see Experimental Section) was performed demonstrating that the quenching effect is only present if the interaction is intact. As a result of the quenching effect, the raw fluorescence was analyzed and used to calculate the affinity. It is unclear if this may have skewed the actual *K*_D_ values for this aptamer.

### 2.2. Principles of Solution-Based Detection Assays for OTA Detection

Given that each of the aptamers displayed relatively similar binding affinity (less than an order of magnitude difference), we next investigated the versatility of each aptamer for incorporation into solution-based detection assays of OTA. We tested two recently described and frequently employed label-free assays, i.e., one colorimetric assay based on the use of gold nanoparticles (AuNPs) and one fluorimetric assays based on the use of commercially available SYBR Green I (SG).

**Figure 3:**
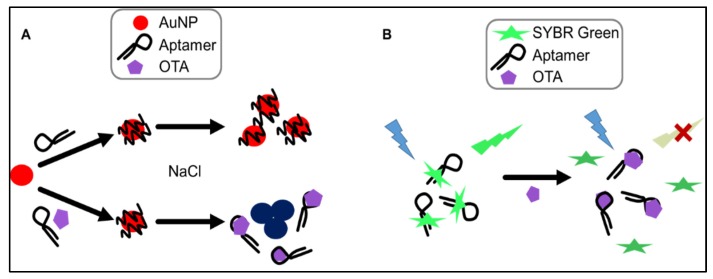
Principle of the gold nanoparticle (AuNP) and SYBR Green I assays (**A** and **B**, respectively) for ochratoxin A (OTA) detection.

The AuNP-based assay makes use of change in optical properties of nanoparticles upon aggregation. In particular, single-stranded DNA (ssDNA) can be used to stabilize AuNPs against salt-induced aggregation by coiling around the AuNP. In the absence of the aptamer’s target (i.e., OTA), the aptamer remains unstructured and coils around the AuNPs. Upon the addition of OTA, the tested aptamers preferentially bind to the mycotoxin, folding into a specific rigid tertiary structure, allowing salt-induced aggregation, and a readily detectable and measurable change in color from red to blue is observed (Figure 3A). This colorimetric assay, using NaCl for salt-aggregation of AuNPs, was reported for the first time by Yang et al. [32] for the detection of OTA in solution in the range of 20 to 625 nM with a limit of detection (LOD) of 20 nM. Recently, another colorimetric assay using AuNPs and a cationic polymer for salt aggregation was reported by Luan et al. [33] for the detection of OTA in 100-fold diluted liquor samples spiked with OTA in the range from 0.05 to 50 ng/mL (0.125 to 125 nM). Both these AuNP assays used the aptamer 1.12.2 for their colorimetric assays.

The second assay we tested was the SYBR Green I (SG) assay. SG is one of the most sensitive dyes available for detecting double strands DNA. SG itself has limited fluorescence but when it interacts with double stranded regions [34] of the aptamer through intercalation, it shows a strong green emission signal (λ_max_ = 520 nm) upon irradiation at 497 nm. When the target is added, the target competes with SYBR Green for binding to the aptamer, leading to significant conformational change of the aptamer and dehybridization of the paired DNA, which would result in the disappearance of fluorescence emission (Figure 3B). Recently, a similar label-free assay was developed using the ultra-sensitive double-strand DNA specific dyes PicoGreen, based on the fact that ssDNA aptamer has the ability to form a double-strand structure with its complementary sequence [35]. This assay was used for the detection of OTA in 100-fold diluted beer sample spiked with OTA in the range from 1 ng/mL to 100 μg/mL (2.5 nM to 250 μM). Also in this case the assay was based on the use of the aptamer 1.12.2.

### 2.3. Comparison of OTA Aptamers Capability for Gold Nanoparticle and SYBR Green I Assays

**Figure 4:**
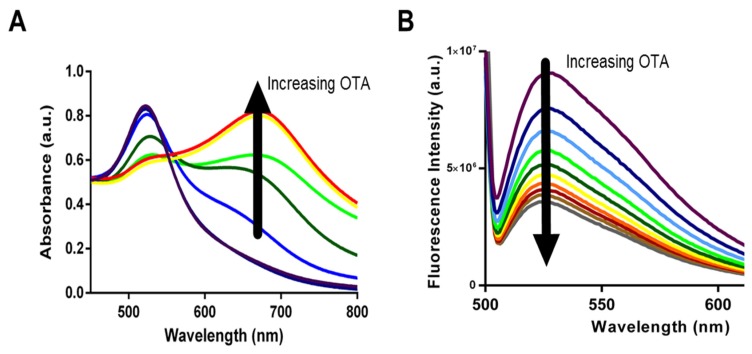
Variation of absorbance (**A**); or fluorescence emission (**B**) signals in relation to different ochratoxin A (OTA) concentrations with aptamer A08min. The gold nanoparticle assay (**A**); and SYBR Green I (**B**) assay. Colors ranges from **purple** (no OTA) to **red**/**brown** (high OTA).

Given that all aptamers tested in the present work displayed similar binding, we sought to compare for the first time the specific analytical capabilities of the aptamers with the two solution-based assays. While aptamers have been frequently compared for their affinity, specificity, and sensitivity to buffer conditions [30] there have been no studies comparing biosensing abilities and limitations. Specifically, we incorporated each aptamer into both AuNP and SG assays and used them for the detection of OTA in solutions containing increasing OTA concentrations from 0.2 nM to 70 nM for AuNP assay and from 200 pM to 70 nM for SYBR Green I assay. Upon addition of OTA, aptamers 1.12.2, A08, and A08min resulted in a significant change in absorbance with the AuNP assay (as example, Figure 4A) and a reduction in fluorescence emission with the SG assay (as example, Figure 4B).

Even though aptamer H8 and H12 have similar binding affinities as 1.12.2, A08, and A08min; there was no response upon addition of OTA when employing aptamer H8 or H12 in the AuNP and SG assays. Given the positive results obtained by incorporating a fluorescein modification on the aptamers used for microscale thermophoresis assay, we repeated both assays using H8 and H12 aptamers with a 5′ fluorescein amidite modifier; however, there was no change in absorbance (AuNP assay) or fluorescence (SG assay). We confirmed this finding using two previously reported solution-based assays, i.e., ultrafiltration and equilibrium dialysis [25,29]. As a result, we proposed that this family of aptamers is not amenable to solution-based detection assays where the aptamer interacts with a competitive dye or nanoparticle surface. These particular aptamers may be very useful for other application [19], but are perhaps not as generally applicable to diverse analytical applications incorporating aptamers.

**Figure 5:**
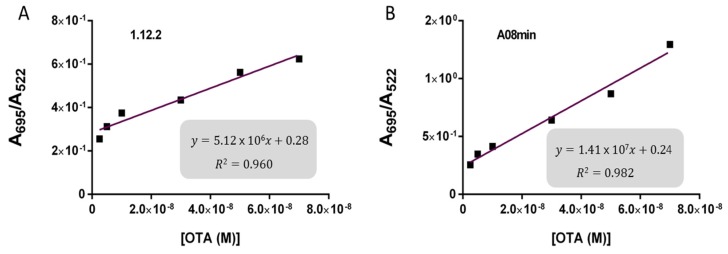
Calibration curves for the AuNP assay. (**A**) 1.12.2 and (**B**) A08min. Linear response was obtained between 24 and 70 nM OTA using aptamer 1.12.2 and between 7 and 70 nM OTA using aptamer A08min.

**Figure 6:**
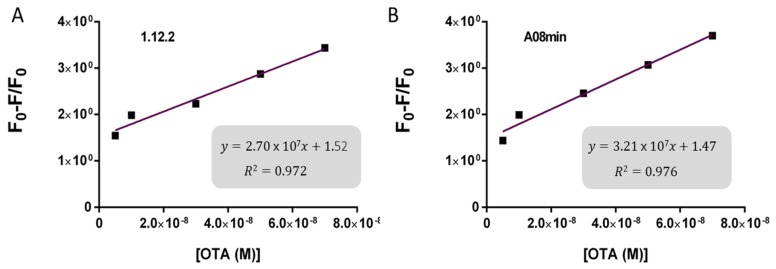
Calibration curves for the SYBR Green I assay. (**A**) 1.12.2 and (**B**) A08min. Linear response was obtained between 5 and 70 nM OTA using aptamer 1.12.2 and between 5 and 70 nM OTA using aptamer A08min.

Based on these findings we decided to focus our attention on aptamers A08min and 1.12.2 to estimate the linear range and LOD values of the two assays and results are reported in Table 2 and graphically shown in Figure 5 for AuNP assay and Figure 6 for SYBR Green I assay.

Both assays showed a good linearity in the range from 2.5 nM to 70 nM OTA for AuNP assay and from 5 nM to 70 nM OTA for SYBR Green I assay. The estimated LOD values ranged from approximately 4 nM for both aptamers used for SYBR Green I assay to 24 nM for aptamer 1.12.2 used for the AuNP assay. Our initial hypothesis, and the standing assumption in the field, is that aptamers with higher affinity will lead to improved assay sensitivity, however, there has been no direct comparison examining this effect with aptamers. Given that aptamer 1.12.2 and A08min displayed affinities in the same range (mid-nanomolar), it is not surprising that with these sensitive assays, the linear ranges are similar. Furthermore, aptamer A08min indeed resulted in lower limits of detection compared to 1.12.2. Interestingly, this effect is more subtle in the SG assay compared to the AuNP assay. This further emphasizes that each candidate may have characteristics other than strictly dissociation constant that make an aptamer more amenable to a particular assay or application. The LOD results obtained herein with both assays were in agreement with those reported by Yang et al. [32] and Luan et al. [33] for AuNP assays and by Lv et al. [35] for Pico Green assay. For each assay, the authors reported higher linear ranges. For example, Yang et al. report up to 625 nM of OTA [32] and Lv et al. [35] up to 100 μM. The established limits of OTA from 0.5 to 20 μg/kg depending on the food matrix correspond to approximately 1.2 to 50 nM OTA. Thus, these larger linear ranges are not required and we did not test higher concentrations.

### 2.4. Implications for Future Aptamer Biosensing

Our work first highlights that each aptamer must be carefully tested within the assay platform of choice. Not only are some aptamers not generally applicable (and may lose function under certain conditions); but the inherent binding affinity is clearly not the only factor affecting the performance of an aptamer in a given platform. This very large effect suggests to us that other conditions must be thoroughly tested and compared to make conclusions about aptamer performance, for example, matrix effects, molecular crowding, pH, and cross-reactivity. Typically, the goal of a selection experiment is to obtain aptamers with the highest affinity possible, however, our results highlight that new aptamer candidates with varying affinity may also be very beneficial (i.e., while 1.12.2 displayed five-fold less affinity compared to A08min according to MST, its performance in the SG assay was comparable). An additional exciting potential for having multiple aptamers with varying affinities is that their inclusion within a sensing platform may increase the linear range. Furthermore, the use of multiple aptamers could allow for robust sensing in a variety of environments, for example if certain sequences are more or less impacted by salinity or pH values. Researchers strive to unmask the very rare sequences with the highest affinity—and often with small molecules, this is a large challenge. Often, selections are deemed “failures” if only high micromolar sequences are uncovered. However, in combination with the multiple strategies for signal enhancement, our results highlight the vast opportunities for applying aptamers with only moderate affinities. Therefore, all novel aptamers should be equally considered for integration into biorecognition platforms.

## 3. Conclusions

In summary, we performed the first head-to-head comparison of the three OTA-binding aptamer families for their ability to recognize and detect OTA in-solution-based assays. Based on our results, we can confidently recommend aptamer A08min as an ideal aptamer candidate for in-solution assays. Not only is our work immediately impactful to researchers aiming to develop new biosensors with OTA aptamers, but it also emphasizes the importance of selecting, screening, and functional verification for the application of interest for any aptamer-target prior to implementation into a new biorecognition assay or application. Future work will examine the cross-reactivity and sensitivity of these aptamers in real samples, and include reference materials for further validation and comparison.

## 4. Materials and Methods

### 4.1. Materials

Ochratoxin A (OTA) standard, chemicals and solvents were reagent grade or better and were purchased from Sigma-Aldrich (St. Louis, MO, USA). Ultrapure water was obtained from a Millipore Milli-Q deionized water system at 18 MΩ (Waters, Milford, MA, USA). Amicon-Ultra (0.5 mL 3000 Da) centrifugal filter units were purchased from Fisher Scientific Canada (Ottawa, ON, Canada). Spin-X centrifuge tube filters (0.22 μm cellulose acetate) were from Corning Incorporated (Corning, NY, USA). All molecular biology grade electrophoresis chemicals were purchased from BioShop Canada (Burlington, ON, Canada). OTA stock solution, with a final concentration of 2.47 mM was prepared by dissolving the solid toxin in toluene/acetic acid 99:1 (*v*/*v*). This standard solution was dried and reconstituted in working buffer or water to obtain appropriate OTA dilutions as described below.

### 4.2. Aptamer Synthesis

The ssDNA aptamer sequences containing the appropriate modifications (Table 1) were prepared using standard phosphoramidite chemistry on a Bioautomation Mermade 6 (Plano, TX, USA). Phosphoramidites, modifiers, activator, deblock, capping, and oxidizing reagents were obtained from Glen Research (Sterling, VA, USA). Standard support columns and acetonitrile were purchased from BioAutomation (Plano, TX, USA). Ultra High Purity 5.0 argon was purchased from Praxair Canada (Mississauga, ON, Canada). DNA was purified with a denaturing polyacrylamide gel electrophoresis (12%) followed by clean-up with Amicon-Ultra (0.5 mL 3000 Da) centrifugal filter units. Sequence synthesis was verified through molecular weight verification using electrospray ionization (ESI) mass spectrometry (Novatia LLC, Monmouth Junction, NJ, USA). Each aptamer investigated was dissolved in Milli-Q deionized water at 18.2 MΩ water to obtain stock solutions and properly diluted in binding buffer prior to use.

### 4.3. Microscale Thermophoresis

Experiments were performed by 2bind Gmbh as described previously [31]. Briefly, 4 μL of each serial dilution of OTA in the appropriate binding buffer were mixed with 4 μL of 20 nM of 5′FAM-labelled aptamer. The final concentrations of OTA ranged from 1.22 nM to 40 μM. The samples were analyzed on a Monolith NT at 25 °C, with 20% LED power and 80% Laser power. To test the nature of the fluorescence quenching effect, a denaturation test was performed. The highest and the lowest concentrations of the ligand (1.22 nM and 40 μM) were mixed with 20 nM labelled protein. The samples were split into two parts. One part remained untreated, the other part was supplied with total 2% SDS, 50% formamide and denatured at 95 °C for 10 min. After placing the denatured samples on ice and diluting the untreated samples with buffer to the same concentration as the denatured samples, the samples were analyzed by their fluorescence. NanoTemper analysis software was used to determine the aptamer *K*_D_ using the fit function from the law of mass action.

### 4.4. Gold Nanoparticle Assay

The gold nanoparticle (AuNP) assay was performed as described previously with minor modifications [36]. All glassware used for AuNP synthesis was cleaned by soaking in aqua regia (3:1 mixture of concentrated HCl/HNO_3_) for 15 min followed by thorough rinsing with deionized water. A volume of 98 mL of deionized water and 2 mL of 50 mM HAuCl_4_ was mixed for a final concentration of 1 mM HAuCl_4_. The solution was heated to boiling with magnetic stirring. Upon boiling, 10 mL of 38.8 mM sodium citrate was added. Heating was continued for an additional 20 min following a change in suspension color to red. The flask was removed from the heat and allowed to cool to room temperature with continued stirring. Nanoparticles were characterized by UV-Visible spectrometry, displaying a λ_max_ = 520 nm.

Samples of OTA in water were prepared at concentrations ranging from 0.2 nM to 70 nM. An aliquot of 6 μL of aptamer (10 μM in water) and 135 μL of the AuNP solution (11 nM) were incubated for 30 min. An aliquot of 243 μL OTA was added into microcentrifuge tubes containing the AuNPs and aptamer and vortexed briefly followed by a 30 min incubation time. An aliquot of 75 μL of 0.5 M NaCl was then added to each microcentrifuge tube and allowed to incubate for a further 5 min. Each sample was then analyzed using UV-Visible spectrometry. The relative absorption ratio between 695 nm and 522 nm (A695/A522) was plotted against OTA concentration. This experiment was performed in triplicate. UV/Vis absorption spectra were obtained using a Cary 300 Bio UV-Visible spectrophotometer (Agilent Technologies, Santa Clara, CA, USA).

The limit of detection (LOD) of the assay was calculated as the mean signal of blank (OTA free) solutions (*n* = 10) plus three standard deviations of the mean signal.

### 4.5. SYBR Green I Assay

This assay was performed as previously described [27]. Briefly, each aptamer, SYBR Green I (SG) and OTA dilutions were prepared in the appropriate buffer reported in Table 1. First, each aptamer was heated to 90 °C for five minutes and cooled to room temperature before use. Four microliters of SG (1×) and four microliters of aptamer (10 μM) were mixed together. OTA varying from 0.2 nM to 70 nM were prepared in the buffer and added directly to the SG-aptamer mixture to a final volume of 125 μL. The fluorescence emission spectra were recorded from 500 to 650 nm using an excitation wavelength of 497 nm. The fluorescence at 525 nm was used to calculate the signal (Equation (1)).

(1) $$Response=\frac{F_{\theta }-F}{F}$$

where $F_{\theta }$ is the fluorescence intensity in the absence of OTA and F is the fluorescence intensity at a given concentration of OTA. Fluorescence was measured with Fluorolog Fluorescence Spectrophotometer with a SpectrAcq controller (Horiba Jobin Yvon, Edison, NJ, USA).

The limit of detection (LOD) of the assay was calculated as the mean signal of blank (OTA free) solutions (*n* = 10) plus three standard deviations of the mean signal.

### 4.6. Ultrafiltration

The ultrafiltration assay, carried out according to McKeague et al. [22], was applied to the fluorescently labeled H8 and H12 aptamers to evaluate if the presence of the fluorescent label could have improved the binding ability of those aptamers towards OTA. Two different aptamers concentrations (i.e., 0.5 μM and 5 μM) were tested in presence of 50 nM OTA. Each dialysis was carried out in duplicate. A solution containing 50 nM OTA in the absence of aptamer was treated likewise and used as control. An aliquot (50 μL) of the centrifuged fractions containing unbounded OTA were analyzed by HPLC to estimate the fraction of OTA bounded to the aptamer according to McKeague et al. [22].

### 4.7. Equilibrium Dialysis

Binding assays by equilibrium dialysis were performed according to the procedure described by McKeague et al. [22]. Two different aptamers concentrations (i.e., 0.5 μM and 5 μM) were tested in presence of 50 nM OTA. Each dialysis was carried out in duplicate.

After the incubation time, an aliquot (50 μL) of the solutions contained in the loading chamber (containing the complex aptamer-OTA and unbounded OTA) and in the receiving chamber (containing unbound OTA) were analyzed by HPLC to estimate the fraction of OTA bound to the aptamer according to McKeague et al. [22].

## Figures and Tables

**Table 1:** OTA aptamers and buffers used for the binding assay studies.

Aptamer	DNA Sequence (5′→3′)	Buffer
1.12.2	GATCGGGTGTGGGTGGCGTAAAGGGAGCATCGGACA	10 mM HEPES, 120 mM NaCl, 5 mM KCl, 10 mM CaCl_2_, pH 7.0
A08	AGCCTCGTCTGTTCTCCCGGCAGTGTGGGCGAATCTATGCGTACCGTTCGATATCGTGGGGAAGACAAGCAGACGT	10 mM Na_2_HPO_4_, 2 mM KH_2_PO_4_, 2.7 mM KCl, 137 mM NaCl, pH 7.4
A08min	GGCAGTGTGGGCGAATCTATGCGTACCGTTCGATATCGTG	Same as A08
H8	GGGAGGACGAAGCGGAACTGGGTGTGGGGTGATCAAGGGAGTAGACTACAGAAGACACGCCCGACA	10 mM Na_2_HPO_4_, 2 mM KH_2_PO_4_, 2.7 mM KCl, 137 mM NaCl, 1 mM MgCl_2_, pH 7.4
H12	GGGAGGACGAAGCGGAACCGGGTGTGGGTGCCTTGATCCAGGGAGTCTCAGAAGACACGCCCGACA	Same as H8

**Table 2:** Summary of in-solution binding and analytical capabilities of OTA aptamers.

Aptamer (ref)	First Reported	MTS	AuNP		SYBR Green I	
	*K*_D_ (nM)	*K*_D_ (nM)	Limit of Detection (LOD) (nM)	Lin. Range (nM)	LOD (nM)	Lin. Range (nM)
1.12.2 [25]	200	524.8 ± 147.8	24	24–70	4	5–70
A08 [27]	290 ± 150	232.6 ± 80.6	n/a	n/a	n/a	n/a
A08min	n/a	96.5 ± 33.3	7	7–70	3	5–70
H8 [26]	130	416.1 ± 119.0	N.D.	N.D.	N.D.	N.D.
H12 [26]	96	629.5 ± 79.7	N.D.	N.D.	N.D.	N.D.

N.D. = Not detected; n/a = Not tested.
